# Serum Paraoxonase, Arylesterase, and Glutathione-S-Transferase Activities and Oxidative Stress Levels in Patients with Mushroom Poisoning

**DOI:** 10.6061/clinics/2018/e16-550

**Published:** 2018-06-29

**Authors:** Sevki Hakan Eren, Ilhan Korkmaz, Fatma Mutlu Kukul Guven, Yusuf Kenan Tekin, Levent Ozdemir

**Affiliations:** I Department of Emergency Medicine, Medicine Faculty, Gaziantep University, Gaziantep, Turkey; II Department of Emergency Medicine, Medicine Faculty, Cumhuriyet University, Sivas, Turkey; III Department of Public Health, Medicine Faculty, Cumhuriyet University, Sivas, Turkey

**Keywords:** Oxidative Stress, Antioxidant Status, Mushroom Poisoning, Emergency Service, Hospitalization Time

## Abstract

**OBJECTIVES:**

Consumption of toxic species of mushrooms may have detrimental effects and increase oxidative stress. Paraoxonase, arylesterase and glutathione-S-transferase are antioxidants that resist oxidative stress. In this study, we analyzed the changes in these enzymes during intoxication due to mushrooms.

**METHODS:**

The study enrolled 49 adult patients with a diagnosis of mushroom poisoning according to clinical findings and 49 healthy volunteers as the control group. The patients with mild clinical findings were hospitalized due to the possibility that the patient had also eaten the mushrooms and due to clinical findings in the late period, which could be fatal. Paraoxonase, arylesterase, and glutathione-S-transferase concentrations, as well as total antioxidant and oxidant status, were determined in the 49 patients and 49 healthy volunteers by taking blood samples in the emergency department.

**RESULTS:**

While paraoxonase, arylesterase, and total antioxidant status were significantly decreased in the patient group (*p*<0.05), glutathione-S-transferase, total oxidant status and the oxidative stress index were significantly higher (*p*<0.05). There was a positive correlation between the hospitalization time and the oxidative stress index (r=0.752, *p*<0.001), whereas a negative correlation was found with glutathione-S-transferase (r=-0.420, *p*=0.003).

**CONCLUSION:**

We observed a significant decrease in paraoxonase and arylesterase and an increase in glutathione-S-transferase and oxidative stress indexes in patients with mushroom poisoning, indicating that these patients had an oxidative status. In particular, a low total antioxidant status and high oxidative stress index may gain importance in terms of the assessment of hospitalization duration.

## INTRODUCTION

Because they contain nutrients and can easily be obtained from nature, natural mushrooms are commonly consumed all over the world. However, the consumption of some toxic species of mushrooms may cause serious poisoning. Clinically, mushrooms mostly result in gastrointestinal findings and are associated with a good prognosis. Depending on the type of mushroom, different clinical situations involving high mortality rates may occur 1,2.

Paraoxonase (PON) 1 and arylesterase (ARES) are enzymes encoded by the same gene and have similar active centers. PON1, also known as aromatic esterase 1 or serum aryldialkylphosphatase 1, is a hydrolase that can hydrolyze paraoxon, a strong inhibitor of cholinesterases, and detoxify other types of organophosphates. The arylesterase enzyme can detoxify organophosphates such as PON1, but it does not exhibit a similar genetic polymorphism. Although the natural substrates of both enzymes are different, the PON1 enzyme can hydrolyze phenyl acetate, a substrate of ARES; therefore, it exhibits the activities of both ARES and PON 3-6.

The most prominent feature of ARES enzymes is that they are antioxidants. The serum PON1 enzyme is responsible for a portion of ARES 7,8.

The paraoxonase enzyme system consists of three different groups, namely, PON1, PON2 and PON3. Substrate specificities of enzymes have lactonase activity and vary widely. With lactonase activity, PONs become involved in the metabolism of many drugs and drug precursors. They can also recognize arachidonic acid metabolites as substrates. Additionally, phospholipase A2-like acyl-ARES activities of PON1 have been identified. However, PON2 and PON3 do not have PON activity. PON 3 is reported to have low ARES activity 9-11. The liver has the highest levels of PON expression and activity. The basic synthesis of PON1 and PON3 occurs in the liver. After synthesis, some of the enzymes are transferred from the liver to the plasma through a specific transport system 12-14.

Glutathione-S-transferase (GST) is a multi-functional enzyme in humans that is found in many tissues and has a broad activity and specific substrate 15-17.

With this feature, GST performs the task of defense in living organisms exposed to potentially toxic chemicals. GST performs detoxification by neutralizing the electrophilic areas of reduced glutathione, which are related to the sulfhydryl group. The resultant product is a water-soluble mercapturic acid that is removed from the body by urinary excretion 18-21. The GST enzyme system performs the detoxification of not only drugs and chemical agents but also some harmful molecules that occur in lipids and DNA, final products of DNA hydroxy peroxides, and alkenol and endogenous electrophilic components 22.

Antioxidants that aim to prevent the damage caused by free radicals consist of enzymes and non-enzyme molecules. The antioxidant/oxidant status of the body can be evaluated by separately measuring the activity of antioxidant enzymes and the concentration of antioxidant/oxidant molecules; however, the general antioxidant/oxidant status can be evaluated more easily by measuring the overall total antioxidant status (TAS) 23 and overall total oxidant status (TOS) 24. Oxidative stress occurs as a result of excessive oxidant formation in the body, a decrease in the amount of antioxidants or a combination of these situations 4. Factors that increase oxidative stress reduce the activity of some antioxidant enzymes, such as PON1 25. In this study, we analyzed the level of antioxidant enzymes (PON, ARES, and GST) as well as the TAS, TOS, and oxidative stress index (OSI) in patients with mushroom poisoning who were admitted to our clinic. We also investigated the correlation of these enzymes (PON, ARES, and GST) with TAS and TOS levels during the hospitalization period.

## MATERIALS AND METHODS

This study was conducted between January 2013 and January 2014 in 49 patients admitted to our university hospital emergency department with mushroom poisoning and 49 healthy volunteers (total of 98 individuals).

Mushroom poisoning diagnosis was made according to the clinical findings in the patients whose symptoms arose within 0-24 hours after ingestion of wild mushrooms while a mycologist was not available to analyze the gastric contents or the mushroom toxins,. Clinical signs were cholinergic toxidromes, anticholinergic syndrome, central nervous system signs, early onset of gastrointestinal (GI) symptoms (e.g., cramps, vomiting, and increased bowel activity) and delayed GI symptoms such as hepato-renal toxicity. We also excluded diseases associated with the differential diagnosis (acute kidney injury, acute liver failure, adrenal crisis, bacterial gastroenteritis, food allergies, food poisoning, hallucinogen use, hemorrhagic shock, hepatic encephalopathy, hepatorenal syndrome, isoniazid toxicity, pediatric asthma, septic shock, and viral gastroenteritis) based on the patient history and laboratory results.

A 5-ml sample of blood was obtained from the brachial vein within the first hour following admission for measurement of GST, ARES and PON1, placed in a sterile vacuum tube, and sent to the biochemistry laboratory. After the blood was centrifuged, the sample was stored in a refrigerator at -80°C. Measurement of serum paraoxonase and ARES activities.

PON1 enzyme activity was performed with a spectrophotometer by measuring the p-nitrophenol absorbance increase within one minute at an ambient temperature of 25°C and wavelength of 412 nm. P-nitrophenol was obtained by paraoxan hydrolysis. The serum PON1 activity determination was performed by modification of the Eckerson, Furlang, Juretic, and Mackness methods 26. Phenyl-acetate was used as a substrate to measure ARES activity. One unit of ARES activity was determined as the μmol phenol that developed within one minute, and the results were expressed as kU/L 27.

### Measurement of serum total antioxidant status

Serum total antioxidants were measured by the automatic measurement method based on the procedure in which the characteristic color formed by the TAS 2,2′-azino-bis (3-ethylbenzothiazoline-6-sulfonic acid) radical was lightened with the antioxidants in the sample 23. The results are expressed as mmol Trolox equivalents/L.

### Measurement of serum total oxidant status

We determined TOS by the automatic measurement method 24. The method involves the conversion of the ferrous ion-o-dianisidine complex in the sample to a ferric ion. The ferric ion forms a colored complex with xylenol orange in an acidic medium. The intensity of color measured by the spectrophotometer is associated with the total quantity of oxidant molecules in the sample. The measurement was calibrated with hydrogen peroxide (H2O2), and the results are expressed as micro-molar H2O2 equivalents per liter (μmol H2O2 equiv./L).

### Determination of GST activity

The volume was increased to 1000 ml by adding 50 μl of 20 mM GSH to 50 μl of 0.2 M sodium phosphate buffer (pH=6.5) and by adding distilled water to 50 μl of 20 mM CDNB and 50 μl of enzyme extract. One unit of enzyme activity is the amount of enzyme that catalyzes 1 μmol product in 1 minute at 30°C, and the activities were calculated based on the increase in the absorbency against the blank at 340 nm and 30°C 28.

OSI was calculated according to the following formula: OSI: TOS/TASx100.

### Statistical analysis

Continuous variables are expressed as the mean±SD or medians (min-max) in the presence of abnormal distributions, and categorical variables are expressed as percentages. Comparisons between groups of patients were performed using the χ2 test for categorical variables, independent samples t test for normally distributed continuous variables, and Mann-Whitney U test when the distribution was skewed. Correlations were evaluated using the Spearman correlation test. The Kruskal-Wallis test was used for comparison of enzyme levels according to the length of hospital stay. All statistical procedures were performed using SPSS software version 14.0 (SPSS Inc., Chicago, IL, USA). A *p*-value of 0.05 was considered significant.

### Ethics

The study was approved by the Cumhuriyet University Ethics Committee in Sivas, Türkiye (2009-09/85). The study adhered to the tenets of the Declaration of Helsinki.

## RESULTS

The difference between the age groups and genders of the patients enrolled in the study was insignificant (*p*>0.05).

ARES and PON levels were significantly decreased due to the increased oxidative stress, whereas TOS and GST levels in the patient group increased. When the groups were compared in terms of OSI, the difference between the groups was significant (Table 1).

**Figure 1:**
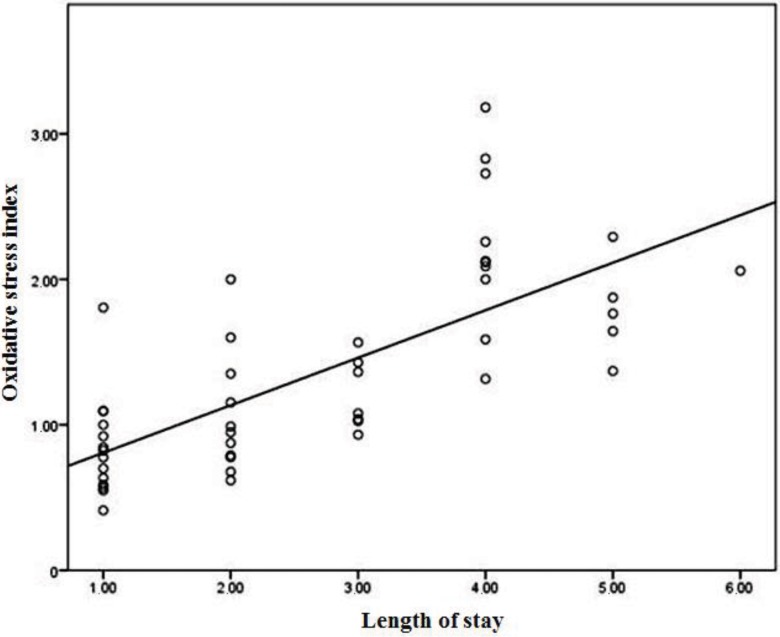
Correlation between the oxidative stress index and length of stay.

**Figure 2:**
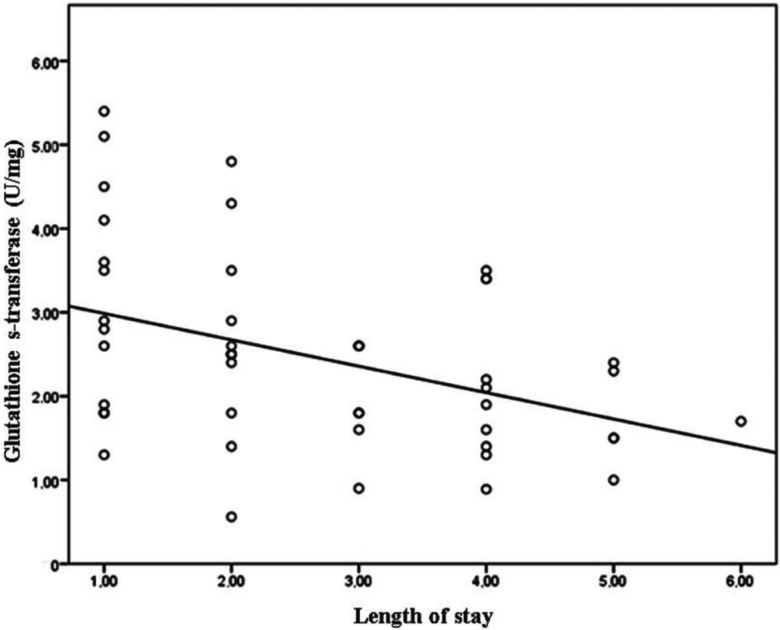
Correlation between GST and length of stay.

When the correlation analysis of TAS, TOS, OSI, GST, ARES and PON levels in the patient group was made based on the length of stay, the differences in the TAS, TOS, OSI, and GST levels were significant, while those in the ARES and PON levels were insignificant (Table 2, Figures 1 and 2).

Length of stay was moderately negatively correlated with GST (r=-0.420, *p*=0.003; Figure 1), TAS (r=-0.548, *p*<0.001), and TOS levels (r=0.435, *p*=0.002) and strongly positively correlated with OSI (r=0.752, *p*<0.001; Figure 2). There was no significant correlation between the length of stay and the other laboratory findings (*p*>0.05).

Enzyme levels according to the length of stay are provided in Table 3.

The duration of hospitalization in patients with lower levels of GST was found to be longer.

## DISCUSSION

PONs are enzymes with antioxidant effects. PON1 shows a protective effect through the detoxification of neurotoxic agents, such as acute organophosphate insecticides and somansarine, and especially against lipid peroxidation, which is very important in atherosclerosis and diabetes pathogenesis. Therefore, it is suggested that increasing the activity and/or concentration of serum PON1 would be useful for the treatment of the disease which increase the oxidative status 29-31. Although the main substrate of PON1 in the human body is unknown, its direct or indirect antioxidant effect seems to be its main effect. PON1 decreases during many infections and inflammation, due to the consumption. Similarly, a reduced PON was observed in relation to the endothelial damage in the eyes of patients with acute silk road disease 32,33. In our study, compared with healthy individuals, we found that PON activity was significantly low in the patients.

The most prominent features of ARES and PON1 are their antioxidant effects. In a study where PON1 and ARES activities in patients with pulmonary hypertension were examined, both enzymes were reduced depending on oxidative stress, and the results are consistent with those of the present study 7.

In another study, compared with the PON and ARES activities in the patients in the control group, those in the patients with multi-trauma were significantly lower, and it was suggested that consideration of these enzymes in diagnosis and treatment could be useful for assessing the severity of the trauma and the potential effectiveness of the treatment 34. Oxidative stress affects PON1 expression and activity 35. Serum PON1 expression decreases with oxidative stress 36, and PON1 even becomes inactivated under oxidative stress 37.

In the study conducted by Eren et al. 2, PON1 enzyme levels of patients with mushroom poisoning were measured at the time of arrival and discharge. The enzyme levels at discharge were found to be higher. The researchers attributed this result to the fact that the enzyme decreased during detoxification during the acute poisoning period and increased again in the following period.

Kati et al. compared the PON, malonyldialdehyde, TAS and ARES levels of 11 patients treated for SSRI poisoning, and while the PON and ARES levels were significantly low, the TAS level was significantly high 38. In our study, consistent with the literature, the PON and ARES levels were significantly lower in the patient group compared with those in the control group. The finding that both enzymes had low levels suggests that they may have been decreased due to their use during the detoxification process. GSTs are an important group of enzymes that eliminate the toxic effects of xenobiotics and electrophilic compounds and thereby prevent alkylation of macromolecules (DNA, RNA, proteins) in cells 39.

GSTs perform the task of detoxification by neutralizing the electrophilic areas of reduced glutathione, which are related to the SH group. In the literature, we did not find any study that investigated the relationship between GST enzymes and intoxication. In some studies, compared with GST enzymes in healthy controls, GST enzymes were significantly higher in patients with complex regional pain syndrome 40. In rats exposed to cold temperatures for a long time, GST enzymes were significantly higher 41.

Oxidative stress is a condition that occurs in many diseases as a result of excessive oxidant formation in the body, a decrease in the amount of antioxidants or a combination of these situations. Antioxidant defense mechanisms of organisms, which consist of many enzymes and antioxidant compounds, work to eliminate oxidants. In instances where antioxidant mechanisms fail, oxidative stress increases and tissue damage occurs. The intake of a high ratio of vitamins A, E and C and antioxidants such as selenium reduces the risk of certain diseases 42-45.

Several studies have shown that elevated OSI levels are associated with inflammatory bowel disease, pemphigus vulgaris, Crimean-Congo hemorrhagic fever, essential thrombocythemia, and various clinical illnesses 46-50.

In our study, the correlation between hospitalization time and PON, ARES, GST, OSİ, TAS and TOS levels was investigated. There was no correlation between PON and ARES levels. A negative correlation was found between TAS and GST, whereas a positive correlation was found between TOS and OSI. TOS and OSI levels measured at the time of the patient’s first arrival may be a poor marker of prognosis. Especially low TAS and high OSI levels may become important factors for assessing hospitalization time.

In conclusion, we observed a significant decrease in PON and ARES and an increase in OSI and GST levels in patients with wild mushroom poisoning, indicating that these patients had an oxidative status. However, these results are not sufficient to explain the relationship between antioxidants and mushroom poisoning. Further studies are required to clarify the possible mechanisms underlying the decreased and increased levels of these factors.

### Limitations

Our study had several limitations. The patients admitted to our emergency department had only mild GI symptoms that occurred in the first 6 hours. We could not identify the mushroom types and toxins, so we could not evaluate the possible differences in antioxidant status with regard to serious cases. Moreover, only PON, ARES, OSI, and GST were examined in our study. An evaluation of more parameters may yield better results and contribute to an increased understanding of the pathophysiology of mushroom poisoning.

## AUTHOR CONTRIBUTIONS

Eren SH and Korkmaz I were responsible for the project development and manuscript writing. Guven FM and Tekin YK were responsible for the manuscript writing. Ozdemir L and Eren SH were responsible for the project development and data collection.

## Figures and Tables

**Table 1:** Baseline characteristics of the study patients.

	Patients (n=49)	Controls (n=49)	*p*
Mean age (years)	47±15	46±13	0.657
Male/Female	27/22	25/24	0.544
TAS	0.8±0.2	0.9±0.3	0.144
TOS	9 (4-18)	7 (3-9)	<0.001
ARES	4421±1292	7162±1985	<0.001
PON	68±24	104±42	<0.001
GST	2.4 (0.6-5.4)	1.6 (2.6-5.0)	0.002
OSI	1.1 (0.4-3.18)	0.8 (0.3-2.5)	<0.001

**Table 2:** Spearman correlation coefficients for the length of stay.

	Length of stay	
	r	*p*-value
TAS	-0.548	<0.001
TOS	0.435	0.002
OSI	0.752	<0.001
GST	-0.420	0.003
ARES	-0.139	0.339
PON	0.042	0.776

**Table 3:** Enzyme levels (mean±SD) according to the length of stay.

	Length of stay (days)				
	1	2	3	4+	*p*
TAS	0.93±0.23	0.89±0.23	0.83±0.21	0.56±0.19	0.00
TOS	7.57±2.90	8.83±3.04	9.71±1.98	11.15±3.39	0.02
OSI	0.84±0.35	1.05±0.41	1.20±0.24	2.08±0.51	0.00
GST	3.08±1.32	2.65±1.16	1.99±.65	2.01±0.83	0.04
ARES	4495.29±1328.11	4650.42±1650.70	4098.14±1041.89	4326.44±1130.82	0.82
PON	67.86±23.48	66.42±26.44	65.86±22.83	69.00±24.29	0.99
